# The conserved microRNA‐229 family controls low‐insulin signaling and dietary restriction induced longevity through interactions with SKN‐1/NRF2

**DOI:** 10.1111/acel.13785

**Published:** 2023-02-07

**Authors:** Latika Matai, Thalyana Stathis, Jonathan D. Lee, Christine Parsons, Tanvi Saxena, Kovi Shlomchik, Frank J. Slack

**Affiliations:** ^1^ HMS Initiative for RNA Medicine, Department of Pathology Beth Israel Deaconess Medical Center, Harvard Medical School Boston Massachusetts USA; ^2^ Department of Molecular, Cellular and Developmental Biology Yale University New Haven Connecticut USA

**Keywords:** aging, dietary restriction, insulin/IGF‐1, microRNA, miR, SKN‐1

## Abstract

Several microRNAs have emerged as regulators of pathways that control aging. For example, miR‐228 is required for normal lifespan and dietary restriction (DR) mediated longevity through interaction with PHA‐4 and SKN‐1 transcription factors in *Caenorhabditis elegans*. miR‐229,64,65, and 66, a cluster of microRNAs located adjacent to each other on chromosome III, are in the same family as miR‐228, albeit with slight differences in the miR‐228 seed sequence. We demonstrate that, in contrast to the anti‐longevity role of miR‐228, the miR‐229‐66 cluster is required for normal *C. elegans* lifespan and for the longevity observed in *mir‐228* mutants. miR‐229‐66 is also critical for lifespan extension observed under DR and reduced insulin signaling (IIS) and by constitutive nuclear SKN‐1. Both DR and low‐IIS upregulate the expression of the miRNA cluster, which is dependent on transcription factors PHA‐4, SKN‐1, and DAF‐16. In turn, the expression of SKN‐1 and DAF‐16 requires *mir‐229*,*64*,*65*,*66*. miR‐229‐66 targets the odd‐skipped‐related transcription factor, *odd‐2* to regulate lifespan. Knockdown of *odd‐2* increases lifespan, suppresses the short lifespan of *mir‐229*,*64*,*65*,*66(nDf63) III* mutants, and alters levels of SKN‐1 in the ASI neurons. Together with SKN‐1, the miRNA cluster also indirectly regulates several genes in the xenobiotic detoxification pathway which increases wild‐type lifespan and significantly rescues the short lifespan of *mir‐229*,*64*,*65*,*66(nDf63) III* mutants. Thus, by interacting with SKN‐1, miR‐229‐66 transduces the effects of DR and low‐IIS in lifespan extension in *C. elegans.* Given that this pathway is conserved, it is possible that a similar mechanism regulates aging in more complex organisms.

## Graphical abstract

miR‐229,64,65,66 family interacts with SKN‐1 to control DR/low‐IIS longevity.

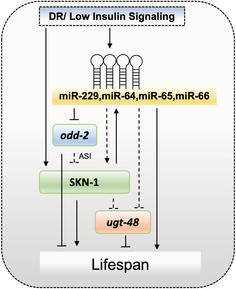

**Abbreviations**
BDR: bacterial dilution regime
CA‐SKN‐1: constitutive nuclear/active SKN‐1
DR: dietary restriction
IIS: insulin signaling
miR: microRNA
OSR: odd skipped related
UTR: untranslated region
WT: wild‐type

## 1 INTRODUCTION

MicroRNAs (miRNAs) are a class of short, noncoding RNAs (ncRNAs) that bind their target messenger RNAs through partial base pair complementarity, resulting in post‐transcription repression of gene expression (Bartel, 2004). Since the discovery of the *lin‐4* and *let‐7* miRNAs regulating developmental timing in *Caenorhabditis elegans*, (Lee et al., 1993; Pasquinelli et al., 2000; Wightman et al., 1993), the last two decades have seen an ascendance in number of findings revealing roles of miRNAs in various aspects of development, metabolism, stress resistance, and aging (Ambros, 2011; Boehm & Slack, 2005; Esquela‐Kerscher & Slack, 2006). It is now evident that multiple miRNAs play a role in regulating lifespan (hereafter referred to as gerontomiRNAs or gerontomiRs), by modulating processes like DNA damage responses, mitochondrial metabolism, and proteostasis (Antebi, 2007; Boehm & Slack, 2005; de Lencastre et al., 2010; Ibáñez‐Ventoso et al., 2008; Kato et al., 2011; Kenyon, 2010).

Research pioneered in *C. elegans* demonstrates that aging is influenced by genetic and regulatory mechanisms that respond to nutrient availability and environmental stress (Kenyon, 2010). Mutations in the insulin/insulin like growth factor (IGF) receptor *daf‐2* lead to lifespan extension and promotes stress resistance in animals (Kimura et al., 1997). This is dependent on key downstream transcription factors DAF‐16, HSF‐1, and SKN‐1 (Ogg et al., 1997; Seo et al., 2013; Tullet et al., 2008), which are translocated to the nucleus to promote the transcription of prolongevity and stress resistance genes. Similarly, animals undergoing dietary restriction also display an extended lifespan and improved stress resistance (Lakowski & Hekimi, 1998), which is dependent on PHA‐4 and SKN‐1 (Panowski et al., 2007) (Bishop & Guarente, 2007). Recent research has begun investigating the role of miRNAs in these longevity paradigms. Notably, several microRNAs exhibit altered expression between wild‐type and *daf‐2* animals (de Lencastre et al., 2010). Among these, miR‐71 functions through the IGF‐1/insulin signaling pathway such that loss of miR‐71 suppresses the long lifespan of *daf‐2* mutant animals. miR‐71 is also required for DR induced longevity in animals, through the interaction with PHA‐4 and SKN‐1 transcription factors (Smith‐Vikos et al., 2014). Several other microRNAs are differentially regulated under these conditions, and further investigation will reveal their role in determining lifespan under these prolongevity pertubations (Kato et al., 2011; Vora et al., 2013; Xu et al., 2019; Zhang et al., 2018; Zhi et al., 2017).

The *C. elegans* genome contain approximately 110 annotated miRNA genes which are grouped into 22 families. Interestingly, certain miRNAs act to promote longevity and stress resistance, while other antagonize it (de Lencastre et al., 2010). For instance, deletion of miR‐71, miR‐246, and miR‐238 decreases lifespan whereas deletion of miR‐239 led to a reproducible and significant extension in lifespan. Interestingly, de Lancastre et al. observed that, miR‐238 and miR239 which belong to the same miRNA family, display opposite effects on lifespan. miR‐239 interacts with insulin signaling pathway to regulate lifespan; however, targets of miR‐238 are still unknown. Such examples of miRNAs within the same family exhibiting opposite phenotypes are rare and not well explored.

We have recently studied the role of miRNA‐228 in regulating adult lifespan where loss of *mir‐228* extends *C. elegans* lifespan, and showed that this miRNA also plays a crucial role in DR mediated longevity (Smith‐Vikos et al., 2014). miR‐229,64,65, and 66 are a cluster of microRNAs localized on chromosome III which are in the same family as miR‐228 and share the same human homologs. Here, we describe the role of miR‐229,64,65,66 in lifespan determination and show that in contrast to its family member miR‐228, this miRNA cluster is critical for promoting adult lifespan. miR‐228 antagonizes the age‐related function of miR‐229,64,65,66 and the double mutant is short‐lived. The miR‐229 cluster is also required for dietary restriction and low‐IIS mediated longevity and is regulated by PHA‐4, SKN‐1, and DAF‐16 transcription factors. In turn, it promotes the expression of SKN‐1 and DAF‐16 and this positive feedback loop between the miRNA cluster and SKN‐1 is a requisite for longevity conferred by constitutive nuclear SKN‐1. The miRNA cluster targets the odd‐skipped‐related (OSR) transcription factor, *odd‐2* to regulate SKN‐1 levels in ASI neurons and lifespan. Further trancriptomic analysis in *mir‐229*,*64*,*65*,*66* mutants suggests that the cluster regulates gene expression in different metabolic, proteolytic, and xenobiotic detoxification pathways and with SKN‐1, regulates gene expression of several xenobiotic detoxification genes in adults, in a manner beneficial for normal lifespan.

## 2 RESULTS

### 2.1 The miR‐229,64,65,66 cluster is required for adult lifespan and health span

**FIGURE 1:**
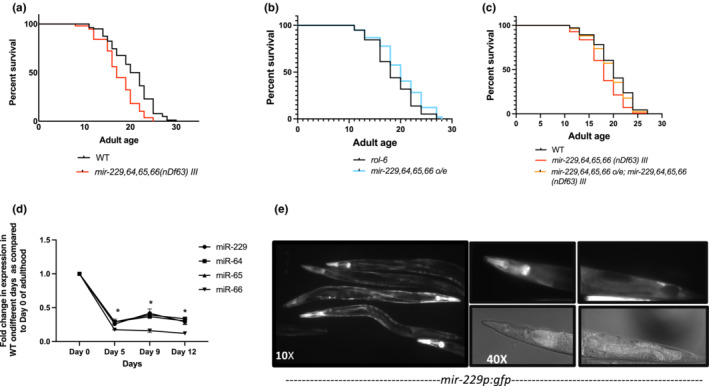
miR‐229,64,65,66 cluster is required for adult lifespan in *Caenorhabditis elegans* (a). Survival curves of lifespan assays in WT and *mir‐229‐66(nDf63) III* mutants indicate that *miR‐229*‐*66(nDf63) III* mutants are short‐lived (*p* < 0.0001 based on log‐rank test). (b) Overexpression (oe) of the *mir‐229*,*64*,*65*,*66* cluster extends longevity (*p* < 0.0001 based on log‐rank test). (c) Survival curves of lifespan assays in WT (N2) and *mir‐229‐66(nDf63) III* mutants overexpressing a WT copy of *mir‐229*,*64*,*65*,*66* indicate that miR‐229,64,65,66 overexpression is sufficient to rescue the short‐lived mutant phenotype. (*p* < 0.0001 based on log‐rank test). (d) The miR‐229,64,65,66 cluster is downregulated during aging. The graph represents the fold changes in mature miRNA levels during multiple timepoints in adulthood as compared to Day 0 (young adult animals) of adulthood and normalized against U18 snoRNA (*N* = 3, error bars show SD, * indicates *p* < 0.05, as. determined by Student's *t*‐test, ns = not‐significant). (e) Expression pattern of *mir‐229p:gfp* in an adult animal. Expression is observed in pharynx, intestine, seam cells, and hypodermis.

We have previously demonstrated the role of miRNA‐228 in antagonizing lifespan where miR‐228 loss of function in *C. elegans* delays aging. miR‐229,64,65,66 are a cluster of 4 miRNAs which are in the same family (based on seed sequence similarity (Figure S2b) as miR‐228 and share the same human homologs (Ibáñez‐Ventoso et al., 2008)). miR‐229,64,65,66 are immediately adjacent to each other on the same chromosome and are thought to share the same promoter (Martinez et al., 2008). These 4 clustered miRNAs also have identical seed sequences to each other, and miR‐64 and 65 have an almost identical mature miRNA sequence. Given this seed conservation, it is predicted that these miRNA would share downstream targets (Ibáñez‐Ventoso et al., 2008). In order to investigate the role of miR‐229,64,65,66 cluster in aging, we performed lifespan analysis in *mir‐229*,*64*,*65*,*66* mutant animals. Interestingly, unlike miR‐228, the loss of *mir‐229‐66* cluster significantly reduces lifespan compared to wild type, suggesting its requirement in maintaining adult lifespan (Figure 1a). These results are in agreement with those of Nehammer et al., 2015, in which the miR‐229,64,65,66 cluster has a lifespan‐related role at high temperature (Nehammer et al., 2015). To explore this further, we generated transgenic lines overexpressing *mir‐229*,*64*,*65*,*66* under its own promoter. A *C. elegans* genomic DNA fragment including the entire *mir‐229*,*64*,*65*,*66* locus, as well as 2 kb upstream and 500 bp downstream was amplified and injected into wild‐type animals. Mature miRNA expression of miR‐229,64,65,66 is upregulated threefold to fourfold in these transgenic animals (Figure S1a). In contrast to the loss of *mir‐229*,*64*,*65*,*66*, overexpression of miR‐229,64,65,66 confers extended longevity, attesting to its positive role in lifespan (Figure 1b). This miRNA cluster alone is responsible for promoting longevity because *mir‐229*,*64*,*65*,*66* overexpression is sufficient to rescue the short‐lived mutant phenotype (Figure 1c).

Recently, we and others have found that miRNAs display a distinct spatiotemporal pattern where most gerontomiRs display a dynamic expression pattern during aging (Boulias & Horvitz, 2012; Brosnan et al., 2021; de Lencastre et al., 2010; Kato et al., 2011; Martinez et al., 2008; Zhou et al., 2019). In order to investigate the age‐associated regulation of miR‐229‐66 cluster, we measured mature miRNA expression of all four miRNAs of the cluster at different times during adulthood. Interestingly, miR‐229,64,65, and 66 exhibit a substantial decline in mature miRNA expression during the first 5 days of adulthood followed by consistent low expression with age (Figure 1d). Since miRNAs are expressed in certain tissues to spatially regulate gene expression and function (Boulias & Horvitz, 2012), we studied the tissue activity of miR‐229,64,65,66 promoter. We generated a transgenic strain expressing *gfp* under the *mir*‐*229* promoter. The *mir‐229* promoter drives the expression of *gfp* in the pharynx, intestine, and hypodermis and seam cells (Figure 1e). Further investigation is required to explore whether expression in these tissues is necessary and sufficient to regulate longevity.

Further, we also found that the longevity and stress response phenotypes of this miRNA cluster are highly correlated, as the short‐lived *mir‐229*,*64*,*65*,*66* mutant animals were more sensitive to heat stress than the wild‐type animals (Figure S1b). These results are in agreement with those of Nehammer et al., 2015, in which the miR‐229,64,65,66 cluster has a lifespan‐related role at high temperature and *mir‐229*,*64*,*65*,*66* mutant animals exhibited an altered heat stress response (Nehammer et al., 2015). To further examine the longevity‐associated phenotypes of *mir‐229*,*64*,*65*,*66* mutants, we examined movement rates (Figure S1c) and rates of autofluorescence accumulation (Figure S1d). While *mir‐228* mutants moved more quickly (Smith‐Vikos et al., 2014), *mir‐229*,*64*,*65*,*66* mutants appeared to move more slowly on a given day compared to wild type (Figure S1c). These results are in agreement with those of Nehammer et al., 2015, in which *mir‐229*,*64*,*65*,*66* mutant animals exhibit altered motility rates. Furthermore, as the animals aged, *mir‐229*,*64*,*65*,*66* mutants accumulate autofluorescence faster compared to wild‐type animals (Figure S1d). This indicates that *mir‐229*,*64*,*65*,*66* mutants are not only short‐lived but also appear to age more quickly than wild‐type animals, that is, in an opposite manner from that of *mir‐228* mutants. Moreover, the short‐lived phenotype is likely not due to unrelated sickness, as the brood size of multiple adult animals was measured and was not significantly different from wild type (Figure S1e).

The miRNA cluster lies within an intron of the *gcn‐1* gene (Nukazuka et al., 2008), and the *mir‐229*,*64*,*65*,*66* mutant also delete the first 21 nts of *gcn‐1* exon 3. *gcn‐1* mutants are also short‐lived (*p* < 0.0001), similar to *mir‐229*,*64*,*65*,*66*; however, overexpression of the miRNA cluster rescues this *gcn‐1* short‐lived phenotype (Figure S1f), indicating that the mutant phenotype was not due to deletion of the host gene, *gcn‐1*. The miRNAs are sufficient to cause the longevity phenotype alone without *gcn‐1*.

### 2.2 miR‐228 antagonizes the effect of miR‐229,64,65,66 on longevity

**FIGURE 2:**
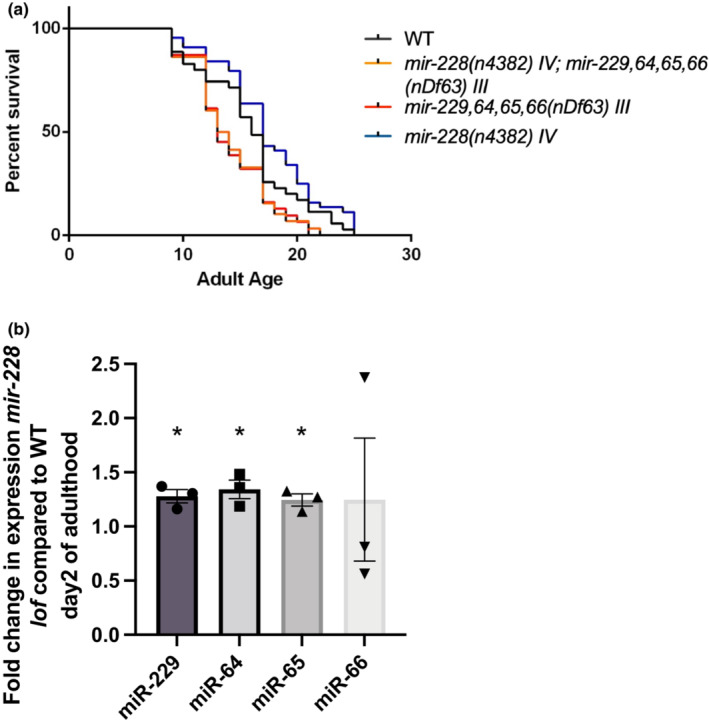
miR‐228 antagonizes miR‐229,64,65,66 during aging. (a) Survival curves of lifespan assays in WT, *mir‐229*,*64*,*65*,*66(nDf63) III*, *mir‐228(n4382) IV and mir‐228(n4382) IV;mir‐229*,*64*,*65*,*66(nDf63) III* double mutants indicate that longevity observed in *mir‐228(n4382) IV* mutant animals is suppressed in a *mir‐228(n4382) IV;mir‐229*,*64*,*65*,*66(nDf63) III* double mutant (*p* < 0.05 via a log‐rank test). (b) Mature miRNA levels of miR‐229,64,65, and 66 are increased in a *mir‐228* mutant background. Graph represents fold changes in mature miRNA levels in *mir‐228(n4382) IV* mutants as compared to WT on Day 2 of adulthood. *N* = 3, error bars show SD, * indicates *p* < 0.05, as determined by Student's *t*‐test, ns = not‐significant.

As miR‐228 antagonizes longevity and miR‐229,64,65,66 promotes longevity, we explored the underlying relationship behind their opposing roles in lifespan regulation. Interestingly, we have found that there may be a direct relationship between miR‐229,64,65,66 and miR‐228. We found that the *mir‐228* mutant requires a wild‐type copy of *mir‐229*,*64*,*65*,*66* in order to be long‐lived, as the double mutant is short‐lived (Figure 2a). Thus, miR‐228 may be acting upstream of and antagonizing miR‐229,64,65,66. This genetic epistasis result is supported by the finding that mature levels of miR‐229,64,65,66 are increased in the *mir‐228* mutant background, indicating that miR‐228 normally functions to repress levels of these miRNAs (Figure 2b). Levels of miR‐228 are also upregulated in the *mir‐229‐66* mutant, indicating a negative feedback loop between these miRNAs (Figure S2a). In order to understand whether these miRNAs regulate similar or different downstream targets, we compared their mature miRNA sequences. Multiple sequence alignment indicated a nucleotide difference in the seed sequence of miR‐228 as compared to miR‐229‐66 family of miRNAs (Figure S2b), which further suggests that these miRNAs likely target different transcripts in regulating lifespan; however, we did not rule out that they might share common targets.

### 2.3 The miR‐229‐66 cluster is required for DR and low insulin signaling mediated longevity

**FIGURE 3:**
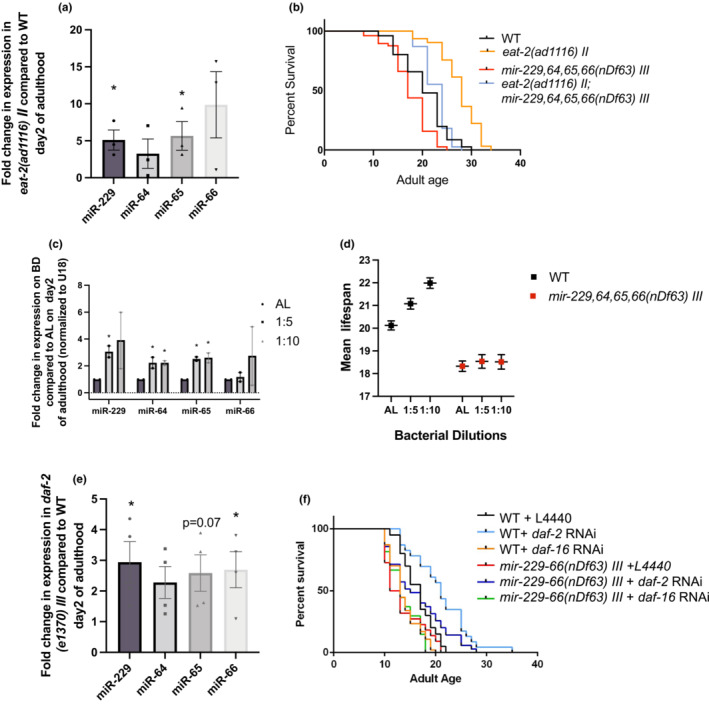
miR‐229,64,65,66 is required for dietary restriction and low‐IIS mediated longevity. (a) DR upregulates miR‐229,64,65,66. The graph represents the fold changes in mature miRNA levels in *eat‐2(ad1116) II* as compared to WT on Day 2 of adulthood (*N* = 3, error bars show SEM, * indicates *p* < 0.05, as. determined by Student's *t*‐test). (b) The lifespan extension observed in an *eat‐2(ad1116) II* mutant is partially suppressed in the *eat‐2;mir‐229*,*64*,*65*,*66* double mutant. Survival curves of lifespan assays in WT, *mir‐229*,*64*,*65*,*66(nDf63) III*, *eat‐2(ad1116) II* and *eat2(ad1116) II;mir‐229*,*64*,*65*,*66(nDf63) III* double mutant (* = *p* < 0.001, based on log‐rank test). (c) The graph represents the fold changes in mature miRNA expression in animals fed on diluted bacteria as compared to ad libitum (*N* = 2, Error bars represent SEM, * indicates *p* < 0.05, as. determined by Student's *t*‐test). (d) miR‐229,64,65,66 is required for longevity conferred by BDR. *mir‐229*,*64*,*65*,*66(nDf63) III* mutants cannot extend lifespan when fed with diluted *E. coli* (OP50) as compared to ad libitum. Graph represents the mean lifespan of WT and *mir‐229*,*64*,*65*,*66(nDf63) III* mutants on ad libitum and diluted bacteria. (e) Low IIS upregulates miR‐229,64,65,66. The graph represents the fold changes in mature miRNA levels in a *daf‐2* mutant as compared to WT on Day 2 of adulthood. *N* = 4, error bars show SEM, * indicates *p* < 0.05, as determined by Student's *t*‐test. (f) Survival curves of lifespan assays in WT and *mir‐229*,*64*,*65*,66 mutant on EV, *daf‐2* and *daf‐16* RNAi indicate that *mir‐229‐66(nDf63) III* cannot extend lifespan on *daf‐2* RNAi as compared to WT and cannot further suppress the short lifespan observed on *daf‐16* knockdown (* = *p* < 0.001, based on log‐rank test).

Dietary restriction (DR) is a conserved intervention that increases lifespan and activates cytoprotective pathways across species (McDonald & Ramsey, 2010). Knocking down the *eat‐2* gene or implementing a bacterial dilution regime (BDR) has been shown to enhance lifespan in adult animals (Lakowski & Hekimi, 1998). Recent research has shown a role and regulation of miRNAs in dietary restriction‐induced longevity (Smith‐Vikos et al., 2014; Vora et al., 2013; Xu et al., 2019). Therefore, we tested if any of miR‐229,64,65 or 66 are differentially regulated under dietary restriction. Interestingly, animals undergoing DR (both *eat‐2* mutant and BDR) upregulated the expression of these miRNAs as compared to wild‐type animals growing ad libitum (Figure 3a,c). Moreover, since miR‐229‐66 is required for healthy adult lifespan, we further investigated the importance of this upregulation by performing lifespan analysis in *eat‐2* mutant animals in the presence and absence of *mir‐229*,*64*,*65*,*66*. Knocking out *mir‐229*,*64*,*65*,*66* partially (yet significantly) suppressed the long lifespan of *eat‐2* animals, indicating the importance of this miRNA cluster in DR mediated longevity (Figure 3b). We also confirmed the well‐established finding that wild‐type animals fed diluted bacteria (BDR) display an extended lifespan, and correspondingly, miR‐229‐66 cluster is required for complete extension of longevity conferred by BDR (Figures 3d and S3a).

Besides dietary restriction, the insulin/IGF‐1 signaling pathway has also evolved to respond to the nutritional status of an organism and in turn affects lifespan. Inhibition of *daf‐2*, the insulin/IGF‐1 receptor has been shown to result in a dramatic increase in lifespan and is dependent on transcription factors DAF‐16, HSF‐1 and SKN‐1 (Kimura et al., 1997; Ogg et al., 1997; Seo et al., 2013; Tullet et al., 2008). Recently, miRNAs have also been found to interact with the insulin signaling pathway to influence lifespan and innate immunity (Zhang et al., 2018; Zhi et al., 2017).

We observed that, like dietary restriction, miR‐229,64,65, and 66 levels are also upregulated in *daf‐2* mutants as compared to wild type (Figure 3e) Additionally, a wild‐type copy of *mir‐229*,*64*,*65*,*66* is essential for longevity observed under *daf‐2* knockdown, as *mir‐229*,*64*,*65*,*66* mutants partially (yet significantly) suppressed the long lifespan observed on *daf‐2* RNAi (Figure 3f). Overall, this indicates that both low‐insulin signaling and dietary restriction employ miR‐229,64,65,66 to bestow lifespan benefits. Although these longevity paradigms were earlier viewed as independent, recent research suggests that these longevity paradigms are intricately connected and miR‐229,64,65,66 cluster forms a potential link between the two to influence lifespan (Greer et al., 2007; Lapierre et al., 2013; Singh et al., 2016; Tullet et al., 2008).

### 2.4 The miR‐229,64,65,66 cluster is regulated by PHA‐4, SKN‐1 and DAF‐16

**FIGURE 4:**
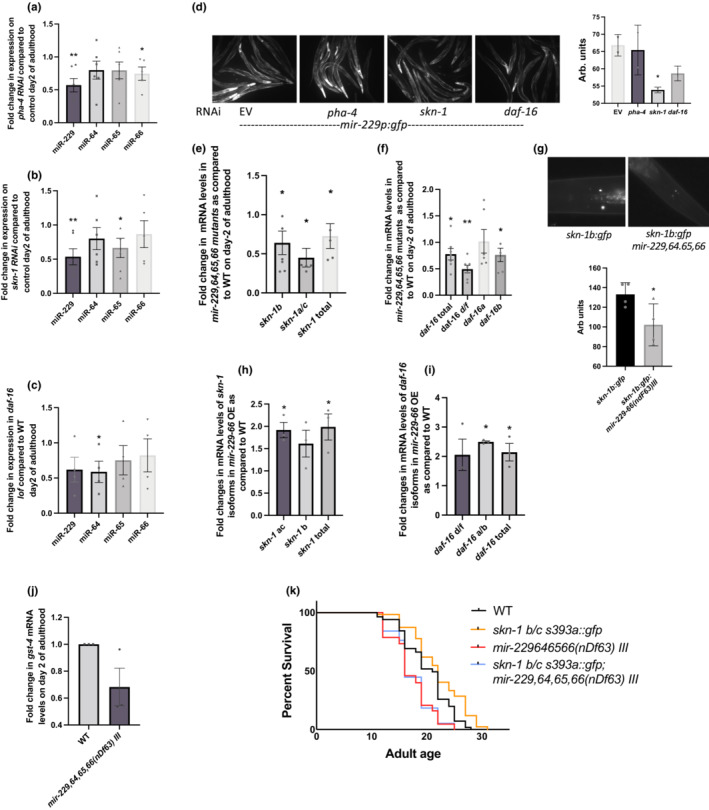
miR‐229 cluster interacts with PHA‐4, SKN‐1 and DAF‐16. PHA‐4, SKN‐1, and DAF‐16 promote the expression of miRNA cluster. Graphs representing fold change in mature miRNA expression of miR‐229,64,65, and 66 on (a) *pha‐4* RNAi, (b) *skn‐1* RNAi and in (c) *a daf‐16* mutant as compared to WT animals on control RNAi on Day 2 of adulthood (*N* = 5,6,4). (d) GFP expression in *mir‐229p:gfp* animals is reduced under *skn‐1* and *daf‐16* knockdown conditions. Representative images of *mir‐229p:gfp* animals on control, *skn‐1* and *daf‐16* RNAi. The graph represents whole body fluorescence averaged over >25 animals, * indicates *p* < 0.05, as. determined by Student's *t*‐test. (e) miR‐229,64,65,66 promotes *skn‐1* expression. The graph represents fold changes in the mRNA levels of all *skn‐1* isoforms in *mir‐229*,*64*,*65*,*66(nDf63) III* mutant animals as compared to WT on Day 2 of adulthood (*N* = 5). (f) mRNA levels of d/f and b isoforms of *daf‐16* decrease in *mir‐229*,*64*,*65*,*66(nDf63) III* mutant animals as compared to WT on Day 2 of adulthood (*N* = 3). (g) SKN‐1B:GFP expression also decreases in ASI neurons in *mir‐229*,*65*,*65*,*66(nDf63) III* mutant as compared to WT. Representative images of the head region of *skn‐1b:gfp* and *skn‐1b:gfp;mir‐229*,*64*,*65*,*66(nDf63) III* animals on Day 2 of adulthood. The graph represents average fluorescence of both ASI neurons for >20 animals (*N* = 4). (h) and (i) mRNA levels of different *skn‐1* (h) and *daf‐16* (i) isoforms increase in transgenic strain overexpressing *mir‐229*,*64*,*65*,*66* as compared to control animals (*N* = 3). (j) Expression of SKN‐1 target *gst‐4* decreases in *mir‐229*,*64*,*65*,*66(nDf63) III* mutant as compared to WT (*N* = 3). For all qPCR experiments, error bars show SEM, * indicates *p* < 0.05, as. determined by Student's *t*‐test (k) Longevity conferred by constitutive nuclear SKN‐1 is suppressed in a *mir‐229‐66(nDf63) III* mutant. Survival curves of lifespan assays of WT, *mir‐229‐66(nDf63)III*, *skn‐1 b/c (s393a):gfp* and *skn‐1 b/c (s393a): gfp*; *mir‐229*,*64.65*,*66(nDf63)III* indicate complete suppression of lifespan extension in *skn‐1 b/c (s393a): gfp;mir‐229*,*64.65*,*66 (nDf63) III* animals (* indicates *p* < 0.001, based on log‐rank test).

Since the miR‐229‐66 cluster is critical for DR and low IIS mediated longevity, we further investigated if this cluster interacts with transcription factors working downstream in these longevity pathways. Therefore, we measured mature miRNA levels of miR‐229,64,65, and 66 in wild‐type animals growing on control, *pha‐4* and *skin‐1* RNAi and in *daf‐16* mutants during early adulthood and observed a downregulation of the miRNAs in all three as compared to wild type on control RNAi (Figure 4a–c). We further explored this regulation at the level of transcription by utilizing the transgenic strain expressing *gfp* under the *mir‐229* promoter. Knocking down *skn‐1* and *daf‐16* significantly reduced the expression of *mir‐229p:gfp;* however, *pha‐4* knockdown had no effect on the *gfp* expression (Figure 4d). This suggest that both SKN‐1 and DAF‐16 promote *mir‐229*,*64*,*65*,*66* transcription, while PHA‐4 only affects mature miRNA levels.

Reanalyzing SKN‐1 CHIP‐seq data at larval stage 3 and 4 from modENCODE database revealed binding in the promoter region of *mir‐229*, suggesting that *mir‐229‐66* might be a direct target of SKN‐1 (Figure S4a). This suggest that under both DR and low IIS, SKN‐1 potentially promotes the transcription of *mir‐229‐66* cluster which plays a critical role in mediating downstream effects towards lifespan extension.

### 2.5 miR‐229‐66 promotes the expression of SKN‐1 and DAF‐16

In order to further investigate the relationship between miR‐229‐66 cluster and these transcription factors, we compared the mRNA expression of *skn‐1*, *pha‐4*, and *daf‐16* between wild‐type and *mir‐229*,*64*,*65*,*66* mutant animals. Interestingly, *skn‐1* (all isoforms) and *daf‐16* (d/f and b isoform) mRNA levels are significantly reduced in *mir‐229*,*64*,*65*,*66* mutants and upregulated in transgenic strain overexpressing miRNA cluster (Figure 4e,f,h,i). However, *pha‐4* levels remain unaffected in these strains (Figure S4b–d). This indicates a positive feedback loop between SKN‐1, DAF‐16, and the miRNA cluster. Consistent with this model, even SKN‐1:GFP expression was downregulated in the ASI neurons in *mir‐229*,*64*,*65*,*66* mutant background (Figure 4g). This in turn downregulates the expression of SKN‐1 and DAF‐16 targets (Figure 4j, S4e); however, a more direct regulation of the targets by miR‐229,64,65,66 is also a possibility. PHA‐4 targets are unaffected in *mir‐229*,*64*,*65*,*66* mutants (Figure S4f). Overall, this hints at a plausible mechanism by which miR‐229‐66 cluster mediates DR and low IIS mediated longevity, that is, by promoting SKN‐1 and DAF‐16 expression. It is also interesting to note that miR‐228 and miR‐229‐66 family of miRNAs regulate *skn‐1* expression in an opposite manner. However, this could be a secondary effect of these miRNAs regulating each other's expression as *skn‐1's* 3'UTR does not contain binding sites for miR‐228 (Figure S4g).

### 2.6 The miR‐229,64,65,66 cluster is required by SKN‐1 to promote longevity

Since both SKN‐1 and miR‐229‐66 cluster seem to play a vital role in delaying aging under DR as well as low insulin signaling, we further explored the importance of this bidirectional interaction between them. To do this, we employed wild‐type transgenic animals carrying a mutant form of SKN‐1[*skn‐1b/c s393a::gfp*] (hereafter sometimes referred as constructive active SKN‐1 or CA:SKN‐1) that constitutively expresses SKN‐1 in intestinal nuclei and displays an extended lifespan (An et al., 2005). A lifespan analysis was performed in wild‐type and *skn‐1b/c(s393a)::gfp* animals in the presence and absence of miR‐229‐66 cluster, and we found that *mir‐229*,*64*,*65*,*66* was required for the long lifespan of *skn‐1b/c(s393a)::gfp* animals. *skn‐1(s393a):gfp;mir‐229*,*64*,*65*,*66* double mutants displayed the lifespan typical for *mir‐229*,*64*,*65*,*66* (Figure 4k). Since lifespan extension of a constitutively active SKN‐1 is dependent on a functional miR‐229‐66 cluster, we believe that the miRNA cluster forms a positive feedback loop with SKN‐1 to mediate downstream effects of these longevity perturbations and appears to be a novel link between DR and low insulin signaling.

### 2.7 The miR‐229,64,65,66 cluster targets *odd‐2* to regulate lifespan

**FIGURE 5:**
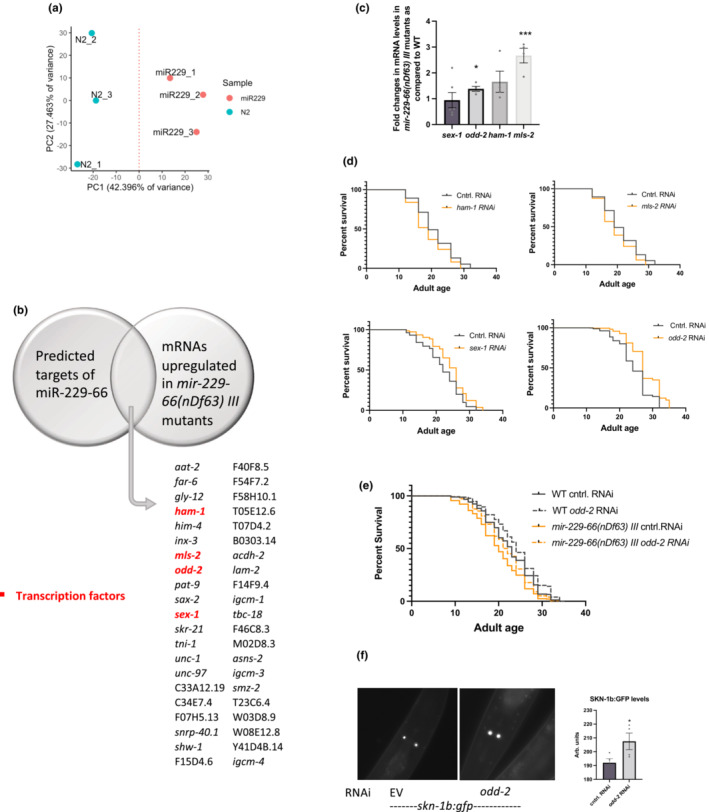
miR‐229‐66 targets homolog of odd‐skipped‐related (OSR) transcription factor, *odd‐2* to regulate lifespan. (a) Principal component analysis of transcriptomic profiling of WT and *mir‐229*,*64*,*65*,*66(nDf63) III* mutants indicate that replicates exhibited high correlations and are clustered together along the principal component 1. (b) A Venn diagram comparing miR‐229‐66 predicted targets (from TargetScan, PicTar and miRanda) with transcripts differentially upregulated in *mir‐229‐66(nDf63) III* mutants as compared to WT during adulthood. (c) The graph represents the fold changes in the expression of *ham‐1*, *mls‐2*, *sex‐1*, and *odd‐2* in *mir‐229*,*64*,*65*,*66(nDf63) III* mutant animals as compared to WT, *N* = 4, error bars show SEM, * indicates *p* < 0.05, as. determined by Student's *t*‐test. (d) Knckdown of *sex‐1* and *odd‐2* increases lifespan. Survival curves of lifespan assays in WT animals on control, *ham‐1*, *mls‐2*, *sex‐1*, and *odd‐2* RNAi (L4 onwards) (* indicates *p* < 0.001, based on log‐rank test). (e) Survival curves of lifespan assays in WT and *mir‐229*,*64*,*65*,*66(nDf63) III* mutants on control and *odd‐2* RNAi (L4 onwards) indicate that suppression of *odd‐2* during adulthood promotes longevity (*p* < 0.0001 based on log‐rank test). (f) ODD‐2 regulates SKN‐1 in ASI neurons. Representative images (left) of the head region of *skn‐1b:gfp* animals on control and *odd‐2* RNAi (L4 onwards) on Day 2 of adulthood. The graph represents the average fluorescence of both ASI neurons for >20 animals (*N* = 4), error bars show SEM, * indicates *p* < 0.05, as determined by Student's *t*‐test.

To better understand the molecular mechanism underlying miR‐229‐66 mediated regulation of lifespan, we performed transcriptomic profiling of wild‐type and *mir‐229*,*64*,*65*,*66* mutants during early adulthood. Principal component analysis suggested that replicates exhibited high correlations and are clustered together along the principal component 1 (Figure 5a). The analysis revealed 1158 genes to be differentially expressed between wild‐type and *mir‐229‐66(nDf63) III* mutant animals during adulthood (Table S1). Further, to identify potential direct targets of miR‐229‐66, we compared the predicted targets of miR‐229‐66 (based on seed sequence complementarity at 3¢UTR) from TargetScan, PicTar, and miRanda with the mRNAs upregulated in *mir‐229‐66* mutants. 41 potential direct target transcripts were found to be upregulated in *mir‐229‐66* mutants during adulthood (Figure 5b) and the list contained 4 transcription factors (TF's) (*ham‐1*, *sex‐1*, *mls‐2*, and *odd‐2*). In order to understand downstream factors that support the role of miR‐229‐66 in lifespan, we chose to further work on these master regulators. qPCR expression analysis revealed significant upregulation of three out of four TF's (*ham‐1*, *mls‐2*, and *odd‐2*); however, the levels of *sex‐1* remain unaffected in *mir‐229‐66* mutant animals (Figure 5c). Further, to understand whether the regulation of these TFs is important for longevity, we performed lifespan analyses under the knock down of each of the three TFs during adulthood. Interestingly, knocking down *odd‐2*, which is the *C. elegans* homolog of odd‐skipped‐related (*osr1*) transcription factor, during adulthood, promoted lifespan extension and also rescued the short lifespan of *mir‐229*,*64*,*65*,*66* mutant (Figure 5d,e). miR‐229‐66 targets the *odd‐2* mRNA 3¢ UTR 56 bp downstream of its stop codon and regulates its expression (Figure S5a). Both *odd‐1* and *odd‐2* in *C. elegans* and the human homologs are known to regulate gut development; however, their role in determining adult lifespan is not yet known (Buckley et al., 2003; Han et al., 2017). The knockdown of *odd‐2* during adulthood also resulted in the upregulation of SKN‐1:GFP in ASI neurons (Figure 5f), something similar to what is observed under DR (Bishop & Guarente, 2007) and consistent with this, *odd‐2* appears to be downregulated under DR (Figure S5c). Overall, this supports the role of miR‐229‐66 in physiological and extended lifespan, that is, by regulating the expression of *odd‐2* and subsequently *skn‐1* during adulthood.

### 2.8 The miR‐229,64,65,66 cluster and SKN‐1 regulate genes in the xenobiotic detoxification pathway to benefit lifespan

**FIGURE 6:**
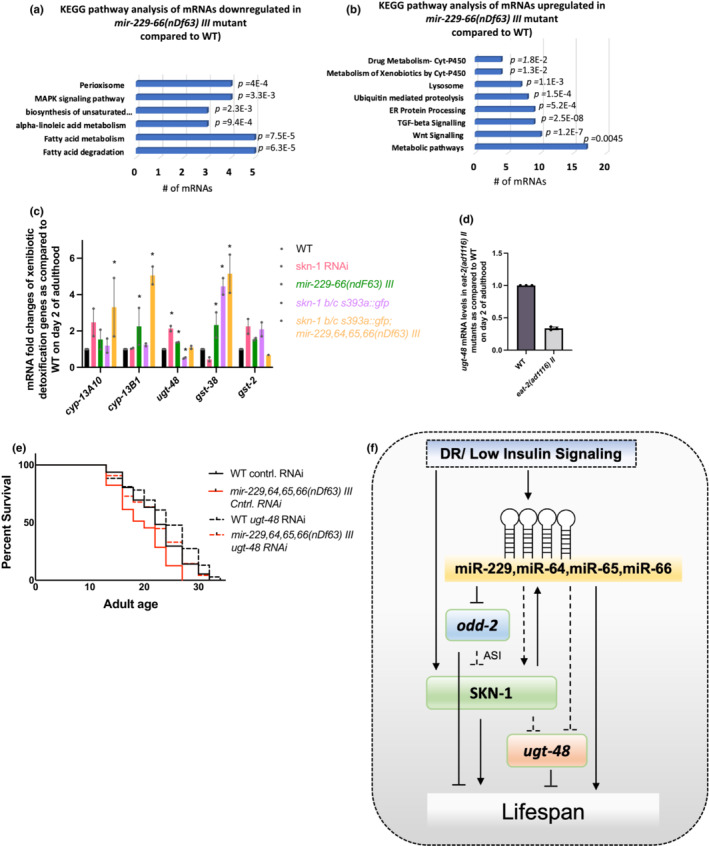
miR‐229‐66 and SKN‐1 regulate xenobiotic detoxification gene *ugt‐48* to regulate lifespan. (a, b) Charts showing GO/KEGG pathway enrichment analysis of differentially expressed genes in *mir‐229‐66(nDf63) III* mutants as compared to WT on Day 2 of adulthood. (c) The graph represents the fold changes in the expression of xenobiotic detoxification genes under *skn‐1* knockdown, *mir‐229‐66(nDf63) III* mutants, constitutive active SKN‐1 (*skn‐1 b/c (s393a):gfp*) and *skn‐1 b/c (s393a):gfp; mir‐229‐66(nDf63) III* double mutants (*N* = 2, Error bars show SEM, * indicates *p* < 0.05, as. determined by Student's *t*‐test, ns = not significant). (d) Expression of *ugt‐48* is downregulated under DR. The graph represents the fold change in *ugt‐48* mRNA levels in *eat‐2(ad1116) II* mutants normalized to WT on Day 2 of adulthood. *N* = 3, error bars show SEM, * indicates *p* < 0.05, as. determined by Student's *t*‐test, ns = not‐significant. (e) Knockdown of *ugt‐48* increases lifespan in WT and *mir‐229‐66(nDf63) III* mutant animals. Survival curves of lifespan assays in WT and *mir‐229*,*64*,*65*,*66(nDf63) III* mutants on control and *ugt‐48* RNAi (L4 onwards) indicate that suppression of *ugt‐48* during adulthood promotes longevity (*p* < 0.001 based on log‐rank test). (f) Working model summarizing the results of the study. Dashed lines indicate likely indirect interactions.

To further explore what downstream processes play a role miR‐229‐66 mediated regulation of adult lifespan, we performed KEGG (Kyoto Encyclopedia of Genes and Genomes) pathway analysis for both up‐ and downregulated genes using the DAVID functional annotation tool (https://david.ncifcrf.gov/) (Figure 6a,b). mRNAs downregulated in *mir‐229‐66(nDf63) III* mutants were significantly enriched in MAPK signaling pathway and fatty acid metabolism, suggesting that miR‐229‐66 directly or indirectly inhibit regulators of these pathways. Interestingly, fatty acid metabolism and degradation have been linked to long lifespan and play a vital role under caloric restriction, thus indicating that promotion of fatty acid metabolism by miR‐229‐66 might contribute to its role in DR mediated longevity (Chamoli et al., 2020). mRNAs upregulated in *mir‐229*,*64*,*65*,*66* mutants were enriched in 9 different pathways (Figure 6b) Importantly, these included TGF‐beta signaling, Wnt signaling, ER protein processing, and ubiquitin mediated proteolysis and contained Skp1 related (Skr) protein which are part of highly conserved SCF‐ubiquitin ligase complex. Additionally, pathways like lysosomal degradation, metabolism, and xenobiotic detoxification by cytochrome P450 were also enriched in the upregulated set, although it is apparent that miR‐229‐66 regulates these pathways indirectly. We validated the sequencing results by qRT‐PCR analysis (Figure S6a–c) which confirmed that mRNAs pertaining to these pathways are indeed upregulated in *mir‐229‐66* mutants as compared to wild‐type. Interestingly, previous studies indicate that ubiquitin mediated proteolysis and xenobiotic detoxification are crucial for promoting DR mediated longevity (Carrano et al., 2009), and the latter is also regulated by SKN‐1 under under physiological and high oxidative stress conditions (Oliveira et al., 2009). Thus, it might be a potential downstream pathway affected by the bidirectional interaction between the miRNA cluster and SKN‐1.

To explore this further, we compared results from the oligonucleotide microarrays from Oliveria RP et.al to identity differentially expressed genes under *skn‐1* knockdown with mRNAs upregulated in *mir‐229*,*64*,*65*,*66* mutants (Oliveira et al., 2009). KEGG pathway analysis of commonly regulated genes also revealed xenobiotic detoxification to be enriched in this dataset (Figure S6e). This included glutathione S‐transferases, UPD‐glucosyl transferases, and cytochrome P450 genes. We further examined their expression levels under SKN‐1 knockdown as well as in the strain constitutively expressing SKN‐1(CA‐SKN‐1) (*skn‐1b/c s393a::gfp*) in nuclei of intestinal cells (Figure 6c). A UPD‐glucosyl transferase‐48 (*ugt‐48*) and glucosyl S‐transferase‐38 (*gst‐38*) showed a significant and opposite regulation under SKN‐1 knockdown and constitutive nuclear expression (Figure 6c). SKN‐1 knockdown during adulthood and CA:SKN‐1 down and upregulated *gst‐38*, respectively, although the levels are kept under check by miR‐229‐66 cluster (Figure 6c). In contrast, the levels of *ugt‐48* are negatively regulated by SKN‐1. It is not surprising because although SKN‐1 in general promotes the activation of different stress defense mechanisms including xenobiotic detoxification, it is also sometimes advantageous to keep some of these genes under check under normal conditions. Correspondingly, the levels of *ugt‐48* were also found to be downregulated in long‐lived *eat‐2* mutants during adulthood, further pointing to its antagonistic role in lifespan regulation (Figure 6d). More interestingly, we found that the inhibition of *ugt‐48* expression by SKN‐1 is dependent on miR‐229‐66 cluster as the levels return to wild type in *skn‐1b/c s393a::gfp*;*mir‐229‐66* double mutants (Figure 6c). The regulation of *ugt‐48* mRNA levels by miR‐229‐66 during early adulthood declines with age as levels of mature miRNAs drastically decrease leading to upregulated *ugt‐48* mRNA levels (Figure S6f).

To further investigate whether the regulation of *ugt‐48* is required to promote longevity, we performed lifespan analysis in wild‐type and *miR‐229‐66* mutants fed on control and *ugt‐48* RNAi. Indeed, we found that knocking down *ugt‐48* during adulthood has a lifespan promoting effect in both wild‐type and *mir‐229‐66* mutants (Figure 5f). A complete rescue of the short lifespan of *mir‐229‐66* mutants under *ugt‐48* knockdown suggests that the miR‐229‐66 mediated regulation of this gene in adult animals is required for normal lifespan. We also checked if *ugt‐48* is regulated by the miR‐229‐66 target ODD‐2; however, the levels of *ugt‐48* mRNA remain unchanged during *odd‐2* knockdown suggesting their role in lifespan regulation may belong to different pathways (Figure S6g). Overall, this supports the benefits of the positive feedback loop between longevity promoting transcription factor SKN‐1 and the geronto‐miR‐229‐66 cluster, which functions to fine tune the expression of these defense genes under physiological conditions in a manner beneficial for lifespan extension (Figure 6f).

## 3 DISCUSSION

In this study, we have uncovered a new role for the miR‐229,64,65,66 family of miRNAs in promoting longevity under physiological and long‐lived conditions. The miR‐229,64,65,66 cluster is highly conserved, and its mammalian homologs have been identified as miR‐425, miR‐200a, and miR‐96/182/183 family (Ibáñez‐Ventoso et al., 2008). Among these, miR‐200a is known to influence aging of human keratinocytes by regulating DNA repair capacity and p16. It also mediates neuroprotection in Alzheimer related deficits and is a potential biomarker (Tinaburri et al., 2018; Wang et al., 2019). miR‐425 is differentially expressed with age, and its deficiency promotes neurodegeneration in Parkinson's disease (Hu et al., 2019; Huan et al., 2018). Given their potential role in aging and age‐related diseases, translation of our findings in mammalian models will help us better understand the mechanism of the human homologs in diseases of aging.

Modulation of the conserved insulin/IGF‐1 signaling pathway or of dietary restriction/caloric restriction leads to the most dramatic effects on lifespan across species. Although these pathways were earlier viewed as independent, recent evidence suggests that relationship between them is more complicated than previously understood. Lifespan extension observed in IIS signaling mutants is strictly dependent on DAF‐16 and is completely independent of PHA‐4 (Lin et al., 2001; Panowski et al., 2007). However, DAF‐16 is required under certain types of DR, for instance, dilution of bacteria on solid media (Greer et al., 2007). Additionally, these pathways have developed extensive mechanisms of crosstalk involving other transcription factors. A DAF‐16 co‐regulator SMK‐1 is required for DR mediated longevity and TFEB homolog HLH‐30 and NRF‐2 homolog SKN‐1 function downstream of both longevity paradigms (Bishop & Guarente, 2007; Lapierre et al., 2013; Tullet et al., 2008). A chromatin modifier *zfp‐1* is also employed by both longevity perturbations to control the expression of overlapping set of genes for promoting lifespan (Singh et al., 2016). Here, we identified a miRNA cluster miR‐229,64,65,66 that interacts with PHA‐4, DAF‐16 and SKN‐1 and consequently is required for extended longevity under both DR and reduced insulin signaling. The two different sensor pathways converge on this miRNA cluster that forms an apparent feed forward loop with SKN‐1 to fine tune the expression of genes in a manner beneficial for lifespan extension.

We have shown the miR‐229,64,65,66 and miR‐228, which belong to the same miRNA family, have opposite effects on wild‐type lifespan where miR‐228 is epistatic to miR‐229,64,65,66. Both miRNAs are required for DR mediated longevity (but in opposite directions), and they exert opposite effects on SKN‐1 expression. miR‐229‐66 positively regulates *skn‐1* mRNA levels while miR‐228 suppresses it (Smith‐Vikos et al., 2014). Multiple sequence alignment indicates that these miRNAs contain either weak or no binding sites in the *skn‐1* 3¢ UTR; therefore, this regulation is likely indirect. It is also interesting to note that both miRNAs negatively regulate each other's expression and this could be due to feedback loops in which both miR‐228 and miR‐229,64,65,66 are regulating *skn‐1* or a direct interaction between them is also possible. This in turn might regulate expression of SKN‐1 and other miR‐229‐66 targets in the respective miRNA mutants.

Our results indicate that miR‐229 potentially targets the ortholog of human odd‐skipped related transcription factor (*osr1)*, *odd‐2* to regulate lifespan. Knockdown of *odd‐2* during adulthood promotes lifespan extension in both WT and *mir‐229‐66* mutants. OSRs (*osr1* and *osr2)* are zinc‐finger transcription factors that are known for their role in gut development in both *C. elegans* and humans (Buckley et al., 2003; Han et al., 2017). They were also recently studied for their role as tumor suppressors in different cancers, through multiple signaling pathways (Otani et al., 2014; Wang et al., 2020; Zhang & Jiang, 2020). Here, we identify a novel role of the OSR homolog *odd‐2* in *C. elegans*, that is, determination of normal adult lifespan and regulation of SKN‐1 expression in the key DR‐mediating neuron, ASI. It will be interesting to identify additional downstream factors regulated by this transcription factor and determining its evolution as a pro‐aging/antisurvival factor in nematodes to a anti‐proliferative factor in humans.

Induction of cytoprotective mechanisms is central for lifespan extension conferred by multiple longevity pathways. Although DR and reduced insulin signaling upregulate most cytoprotective genes, others are kept under check in these longevity perturbations. Example of this include the conserved innate immunity pathway that is regulated by p38 signaling and transcription factor ATF‐7 (Wu et al., 2019). DR downregulates p38‐ATF‐7 immunity to a basal level to achieve complete extension of lifespan. Here, we observed a similar downregulation of the xenobiotic resistance gene *ugt‐48* under DR, and this effect is mediated by SKN‐1 and the miR‐229,64,65,66 cluster. This is not surprising as these innate immunity or detoxification pathways may be advantageous for an organism's defense mechanisms, but not long life and thus are kept suppressed under physiological conditions. Additionally, they might negatively influence the maintenance of cellular and tissue homeostasis, which are important for adult lifespan, providing a rationale behind their regulation during adulthood.

## 4 EXPERIMENTAL PROCEDURES

### 4.1 *Caenorhabditis elegans* strain maintenance

Unless otherwise mentioned, all the *C. elegans* strains were maintained and propagated at 20°C on *E. coli* OP50 using standard procedures. The strains used in this study were as follows: N2 Bristol (wild‐type), *eat‐2(ad1116) II*, *mir‐229*,*64*,*65*,*66(nDf63) III*, *mir‐228(n4382) IV*, *daf‐2(e1370) III*, *daf‐16(mu86) I*, *gpa‐4:skn‐1b:gfp(geIs8)*, *pha‐4:TY1:gfp(wgIs37), skn‐1(b/c)::gfp + rol‐6(su1006) (IdIs7)*.

The above‐mentioned strains were obtained from Caenorhabditis Genetics Centre, University of Minnesota, USA, and were backcrossed 6‐times and cultured using standard protocol. The other strains including *eat‐2(ad1116) II*;*mir‐229*,*64*,*65*,*66(nDf63) III*, *gpa‐4:skn‐1b:gfp(geIs8); mir229*,*64*,*65*,*66(nDf63) III*, *pha‐4:TY1:gfp(wgIs37);mir‐229*,*64*,*65*,*66(nDf63) III* were generated inhouse using standard mating techniques.

### 4.2 Transgenic strains

*mir‐229*,*64*,*65*,*66* overexpression strains were generated as follows: Primers were designed to PCR‐amplify from *C. elegans* genomic DNA fragment including the entire *mir‐229* locus. This PCR amplicon was microinjected (13 ng/μl) into wild‐type animals along with a *rol‐6* (pRF4) as a coinjection marker (50 ng/μl). The *rol‐6* injection marker (50 ng/μl) was also separately microinjected in wild‐type animals as a control. Two independent lines were isolated, and both were tested for miR‐229‐66 expression. *mir‐229p:gfp* strain were generated by cloning a 2 kb region upstream of *mir‐229* locus in the MCS of pPD95.75 and injecting the recombinant plasmid in wild‐type animals at a concentration of 15 ng/μl along with *rol‐6(*pRF4) as the coinjection marker (50 ng/μl).

### 4.3 Dietary restriction assays

Beginning with an OD600 of 0.5, *E. coli* OP50 was diluted 1:5 and 1:10 using M9 buffer to prevent additional bacterial growth. 300 μL of OP50 was pipetted onto standard NGM plates, in which 3 plates per dilution condition and 3 plates of ad libitum (nondiluted OP50) were used. L1‐starved synchronized wild‐type animals were grown to young adulthood on standard NGM plates and then transferred as young adults to ad libitum and diluted OP50 plates. 5‐fluorodeoxyuridine (FUDR) (0.1 g/mL) was added to the plates at adulthood to prevent progeny production. Later, animals were either subjected to lifespan analysis or collected for RNA extraction on Day 2 of adulthood.

### 4.4 Lifespan analysis

Gravid adult animals were bleached, and eggs were hatched on *E. coli* OP50 plates. On reaching adulthood, 50–60 young adult animals were transferred to the RNAi or diluted OP50 plates in triplicates. 5‐fluorodeoxyuridine (FUDR) (0.1 g/mL) was added to the plates to prevent progeny production. At the 7th day of adulthood, sick, sluggish and slow moving animals were removed from the lifespan population, and the remaining animals were considered as subjects for lifespan analysis. Following this, the number of dead animals was scored every alternate day and plotted as % survival against the number of days. Statistical analysis for survival was conducted using Mantel–Cox Log Rank test using Oasis software available at (http://sbi.postech.ac.kr/oasis). The average life span was also determined using the same method and represented as Mean life span ± Standard Error Mean (SEM).

### 4.5 Quantitative RT‐PCR analysis

Total RNA was isolated from animal pellets using Trizol®. Total RNA concentration was measured using the NanoDrop Spectrophotometer (ND‐1000 Spectrophotometer). miRNA expression levels were determined by quantitative RT‐PCR (qRTPCR) using TaqMan miRNA Assays (Applied Biosystems; Chen et al., 2005). Assays specific to mature miRNAs were tested as per the manufacturer's published protocols. miRNA expression levels were normalized to endogenous control U18. The expression of *pha‐4*, *daf‐16*, or *skn‐1, ugt‐48 and odd‐2* mRNA was assayed using SYBR Green I according to manufacturer protocols (Roche). The expression levels were normalized to mRNA levels of the *act‐1* gene. qPCR primers were designed to span at least one exon‐exon boundary. Statistical significance was determined using Student's *t‐*test.

### 4.6 modENCODE TF‐ChIP data analysis

FASTQ reads from modENCODE skn‐1 ChIP‐seq datasets were downloaded from GEO with accession numbers SRR947366, SRR947367, SRR947368, and SRR947369 for L3 animals and SRR849697, SRR849698, SRR849699, and SRR849700 for L4 animals. Reads were aligned to the WBcel235 reference genome using bowtie2 v2.2.9 with options “‐‐local ‐‐very‐sensitive‐local ‐‐no‐unal”. Duplicate reads were removed with Picard v2.8.0.

Visualization was performed by mapping insertions to a genome‐wide sliding 150 bp window with 20 bp offsets with bedops v2.4.30, followed by conversion to bigwig format with wigToBigWig from UCSC tools v363. Genome tracks were visualized with Integrative Genomics Viewer v2.5.0.

### 4.7 RNAseq and GO‐analysis

L1 synchronized wild‐type and *mir‐229*,*64*,*65*,*66(nDf63) III* animals (in three biological replicates) were grown on OP50 till Day 2 of adulthood and RNA were isolated as mentioned above. Quality control analysis for the isolated RNA was done using Bioanalyzer 2100 RNA 6000 NanoAssay chip (Agilent Technologies, Santa Clara, CA, USA). The cDNA libraries were subsequently constructed by the Kapa Hyper‐prep mRNA capture kit (Roche Inc.) and subjected to sequencing on a NextSeq 500 (75 bp, paired end). FASTQ reads were aligned to the WBcel235 reference transcriptome (ensembl release v103) using salmon v0.14.1 with options “‐‐seqBias ‐‐useVBOpt ‐‐gcBias ‐‐posBias ‐‐numBootstraps 30 ‐validateMappings”. Length‐scaled transcripts per million were acquired using tximport v1.18.0, and differential expression analysis was performed by DESeq2 v1.30.1. Principal component analysis was performed with counts transformed by the varianceStabilizingTransformation function from DESeq2, and shrunken log2 fold changes were determined with DESeq2. Raw RNA‐seq datasets are available upon reasonable request.

### 4.8 Heat stress assay

L1‐starved synchronized animals were reared on standard NGM plates until Day 1 adulthood. 30 animals were then transferred to each plate containing FUDR, and 3 plates were used per strain. Mutant and wild‐type animals were simultaneously exposed to a 4‐h heat shock in a 35°C incubator, and animals were allowed to recover overnight by transferring plates to 20°C. The number of surviving animals on each plate was then recorded. Survival was scored by gently prodding each animal with a platinum wire. Mean and standard error were determined from experiments performed in triplicate. *p*‐values were calculated via Student's *t‐*test.

### 4.9 Brood size assay

Fertility was measured by transferring five wild‐type and five *miR‐22*,*966(nDf63) III* animals post‐L4 molt (~56 h) to individual plates every day and counting the number of eggs that were laid for each animal. The starting and end points of reproduction were similar for all animals observed. Statistical significance was determined using Student's *t‐*test.

### 4.10 Measurement of SKN‐1B:GFP and PHA‐4:GFP levels

Transgenic animals expressing SKN‐1B:GFP or PHA‐4:GFP [*gpa‐4:skn‐1b:gfp(geIs8)*, *pha‐4:TY1:gfp(wgIs37)*, *gpa‐4:skn1b:gfp(geIs8);mir‐229*,*64*,*65*,*66(nDf63) III*, *pha‐4:TY1:gfp(wgIs37);mir‐229*,*64*,*65*,*66(nDf63) III*] were bleached, and eggs were hatched on OP50 plates. Similarly, skn‐1(b/c)::gfp + rol‐6(su1006) (IdIs7) and animals were hatched and grown on OP50 till L4, after which they were transferred on respective RNAi plates. Fifty Day 2 gravid adult animals were immobilized on glass slides coated with 2% agarose using 5 mM sodium azide and visualized under Axio‐imager M2 epifluorescent microscope (Carl Zeiss, Germany) equipped with a monochromatic camera lens (MRm) and GFP filter set. Fluorescence of ≥20 animals over ASI neurons (SKN‐1B:GFP) or whole body (PHA‐4:GFP) was quantified using NIH Image J software and represented as arbitrary units (AU).

## AUTHOR CONTRIBUTIONS

LM, TS, and FS designed research; LM, TS, JDL, CP, TS, and MS performed research; LM and JDL analyzed data; and LM, TS, ad FS wrote the paper.

## CONFLICT OF INTEREST

The authors declare no conflict of interest.

## Supporting information


Figure S1.

Table S1.

Table S2.

## Data Availability

The data that support the findings of this study will be available upon request.
