# Supplementary material for: The conserved microRNA‐229 family controls low‐insulin signaling and dietary restriction induced longevity through interactions with SKN‐1/NRF2
Source: Aging Cell. 2023 Feb 7;22(4):e13785. doi: 10.1111/acel.13785 (PMC10086521; doi:10.1111/acel.13785)
Supplement: Supplementary file 1 — Figure S1. [file ACEL-22-e13785-s003.pdf]

Supplementary information

Supplementary Figure Legends

Figure S1. miR-229,64,65,66 cluster is required for adult lifespan and health-span in *C. elegans* (a) Graph represents fold changes in mature miRNA levels in transgenic strain overexpressing *mir-229,64,65,66* (left) and *mir-229,64,65,66* OE crossed with *mir-229,64,65,66(nDF63) III* (right) and as compared to WT. N=2, error bars show SEM, * indicates $p<0.05$, as determined by student's *t*-test, ns=not-significant. (b) *mir-229,64,65,66 (nDf63) III* mutants are susceptible to heat stress. Graph represents survival percentage of day 1 adults after a 4-hour heat shock at 35 °C. *mir-229,64,65,66 (nDf63) III* mutant animals exhibit lower percentage survival as compared to WT. Error bars represent SD, * indicates $p<0.05$, as. determined by student's *t*-test, ns= not significant. (c) Body bends/minute counted for 25 animals on each day of adulthood depict decreased body bends in *mir-229,64,65,66(nDf63) III* mutant animals as compared to WT with age. (d) miRNA mutants exhibit faster accumulation of body autofluorescence compared to WT. Body autofluorescence, was measured by measuring relative GFP fluorescence using an Axioimager fluorescent microscope over different days of adulthood. (e) miRNA mutants exhibit wild-type brood size. Error bars represent SD, * indicates $p<0.05$, as. determined by student's *t*-test, ns= not significant. (f) miR-229,64,65,66 cluster rescues *gcn-1* short-lived phenotype. Survival curves of lifespan assays in WT and *gcn-1* mutants overexpressing a WT copy of *mir-229,64,65,66* cluster ($p < 0.0001$) indicating that the miRNA cluster is sufficient to rescue the short-lived phenotype of *gcn-1*.

Figure S2: (a) Mature miRNA levels of miR-228 are increased in *mir-229,64,65,66(nDf63) III* mutant animals. Graph represents fold changes in mature miRNA levels in *mir-229-66* mutants as compared to WT on day 2 of adulthood. N=3, error bars show SD, * indicates $p<0.05$, as determined by student's *t*-test, ns=not-significant. (b) Jalview of the multiple sequence alignment representing percentage identity between the mature miRNA sequences of miR-228-5p, miR-229-5p, miR-64-5p, miR-65-5p and miR-66-5p. miR-229-66 family of miRNA display identical seed sequence which differs on one nucleotide from the miR-228 seed sequence.

Figure S3: miR-229,64,65,66 cluster is required for Dietary restriction and low IIS mediated longevity. (a) miR-229,64,65,66 is required for longevity conferred by BDR. *mir-229,64,65,66(nDf63) III* mutants cannot extend lifespan when fed with diluted *E. coli* (OP50) as compared to to ad-libitum. Survival curves of lifespan assays, in which the average lifespan was plotted in part (3d). (*= $p < 0.05$, based on log-rank test).

Figure S4: miR-229 cluster interacts with PHA-4, SKN-1 and DAF-16 (a) Integrative Genomics Viewer's view of SKN-1/NRF-2 biding peaks on promoter regions of *mir-229-66* in L3 and L4 worm as determined by CHIP-seq analysis of *unc-119(ed3)III;wg178(OP178)* strain. Data mined from MOENCODE and reanalyzed using our bioinformatics pipeline. Red box indicate promoter regions where peaks are observed. (Upper) SKN-1 CHIPseq using anti-GFP antibody, (Lower) input DNA. (b) Graph represents mRNA levels of *pha-4* in WT and *mir-229-66 (nDf63) III* mutants (N=3, error bars show SD, ns=not-significant) (c) PHA-4:GFP expression increases in *mir-229,65,65,66 (nDf63) III* mutant as compared to WT. Representative images of *pha-4:gfp* and *pha-4:gfp;mir-229,64,65,66* worms on day 2 of adulthood. Graph represents average fluorescence over > 20 worms (N=3). (d) *pha-4* levels remain unaffected in *mir-229-66* OE as compared to WT (N=2, error bars show SD, ns=not significant). Expression of (e) DAF-16 targets decrease and (f) PHA-4 targets is not affected in *mir-229,64,65,66 (nDf63) III* mutant as compared to WT, (N=9,5), * indicates p<0.05, as. determined by student's t-test, ns=not-significant. (g) Jalview of multiple sequence alignment of miR-228 and miR-229-66 mature miRNA sequences with *skn-1* 3'UTR's potential binding site.

Figure S5. (a) Jalview of multiple sequence alignment of miR-228 and miR-229-66 mature miRNA sequences with *odd-2* 3'UTR's potential binding site. (b) Graph represents fold changes in *odd-2* mRNA levels of in *mir-228(n4382) IV* mutants as compared to WT on day 2 of adulthood. N=4* indicates p<0.05, as. determined by student's t-test, ns=not significant. (c) Graph represents fold changes in *odd-2* mRNA levels of in *eat-2(ad1116) II* as compared to WT on day 2 of adulthood. N=4, ns=not significant. (d) Survival curves of lifespan assays carried out with *rde-1* strains expressing wild-type *rde-1* in intestine, hypodermis and muscle on control and *odd-2* RNAi (ns=not significant, based on log-rank test).

Figure S6: (a) (b) and (c) Graph represents fold changes in mRNA levels for genes pertaining to lysosome, xenobiotic detoxification and metabolic pathway as measured by qRT-PCR in *mir-229,64,65,66 (nDf63) III* mutants as compared to WT on day 2 of adulthood. N=2,* indicates p<0.05, as. determined by student's t-test, ns=not significant. (d) Graph represents fold changes in mRNA levels of xenobiotic detoxification pathway genes in *mir-228* mutants as compared to WT on day 2 of adulthood. N=4,* indicates p<0.05, as. determined by student's t-test, ns=not significant. (e) Microarray analysis for differentially expressed genes under *skn-1* knockdown from Oliveria RP et. al. compared with genes up-regulated in expression in *mir-229,64,65,66 (nDf63) III* mutants as compared to WT followed by KEGG pathway analysis of commonly regulated genes. (f) Graph represents fold change in *ugt-48 mRNA* levels on different days of adulthood. * Indicates p<0.05, as. determined by student's t-test. (g) Graph represents fold change in *ugt-48 mRNA* levels on *odd-2* RNAi as compared to control RNAi, N=5, ns=not significant as determined by student's t-test.

Figure S1

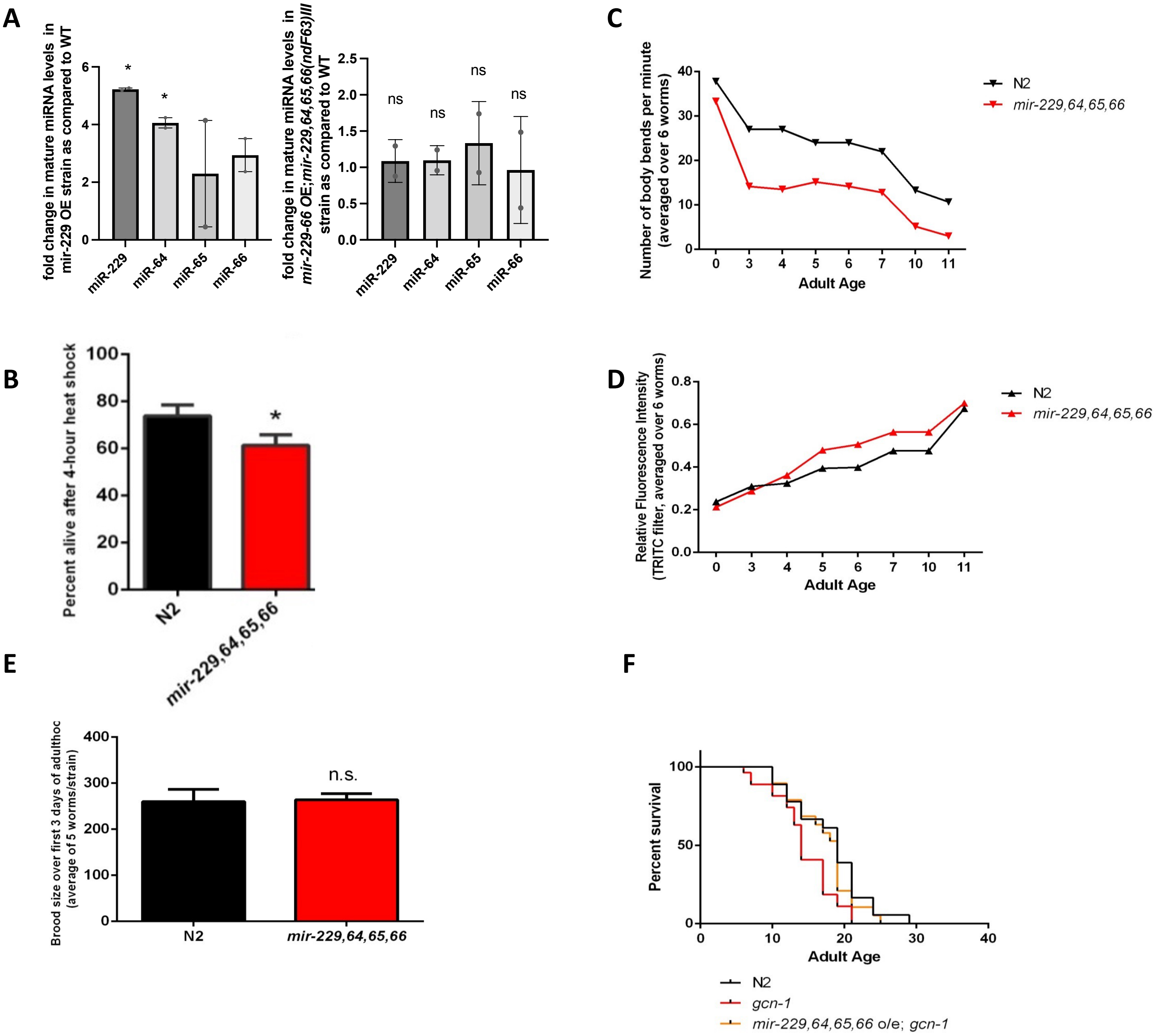
A
fold change in mature miRNA levels in mir-229 OE strain as compared to WT
miR-229
miR-64
miR-65
miR-66
fold change in mature miRNA levels in mir-229-66 OE;mir-229,64,65,66(ndF63)III strain as compared to WT
ns
B
Percent alive after 4-hour heat shock
N2
mir-229,64,65,66
C
Number of body bends per minute (averaged over 6 worms)
Adult Age
N2
mir-229,64,65,66
D
Relative Fluorescence Intensity (TRITC filter, averaged over 6 worms)
Adult Age
N2
mir-229,64,65,66
E
Brood size over first 3 days of adulthoc (average of 5 worms/strain)
n.s.
N2
mir-229,64,65,66
F
Percent survival
Adult Age
N2
gcn-1
mir-229,64,65,66 o/e; gcn-1

Figure S2

A

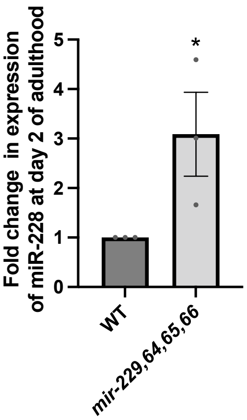

Fold change in expression of miR-228 at day 2 of adulthood
0
1
2
3
4
5
*
WT
mir-229,64,65,66

B

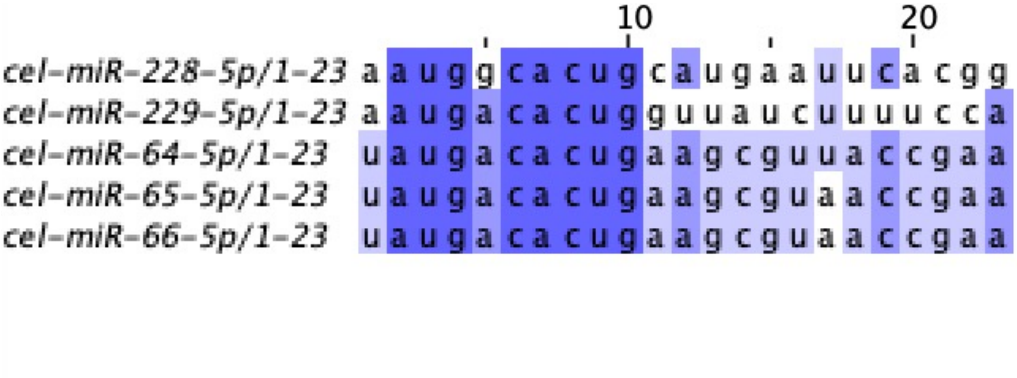

10
20
cel-miR-228-5p/1-23 aauggcacugcaugaauucacgg
cel-miR-229-5p/1-23 aaugacacugguuaucuuuucca
cel-miR-64-5p/1-23 uaugacacugaagcguuaccgaa
cel-miR-65-5p/1-23 uaugacacugaagcguaaccgaa
cel-miR-66-5p/1-23 uaugacacugaagcguaaccgaa

Figure S3

A

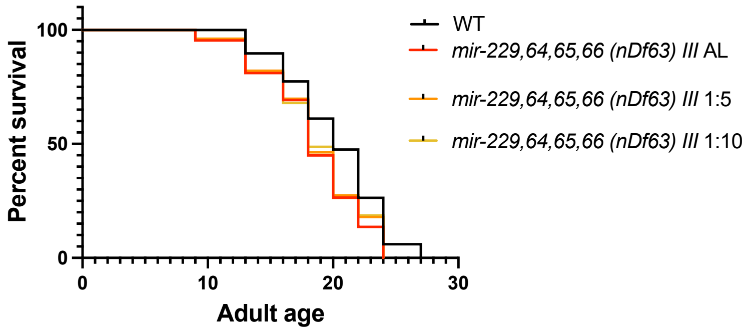

100
50
0
Percent survival
0
10
20
30
Adult age
WT
mir-229,64,65,66 (nDf63) III AL
mir-229,64,65,66 (nDf63) III 1:5
mir-229,64,65,66 (nDf63) III 1:10

Figure S4

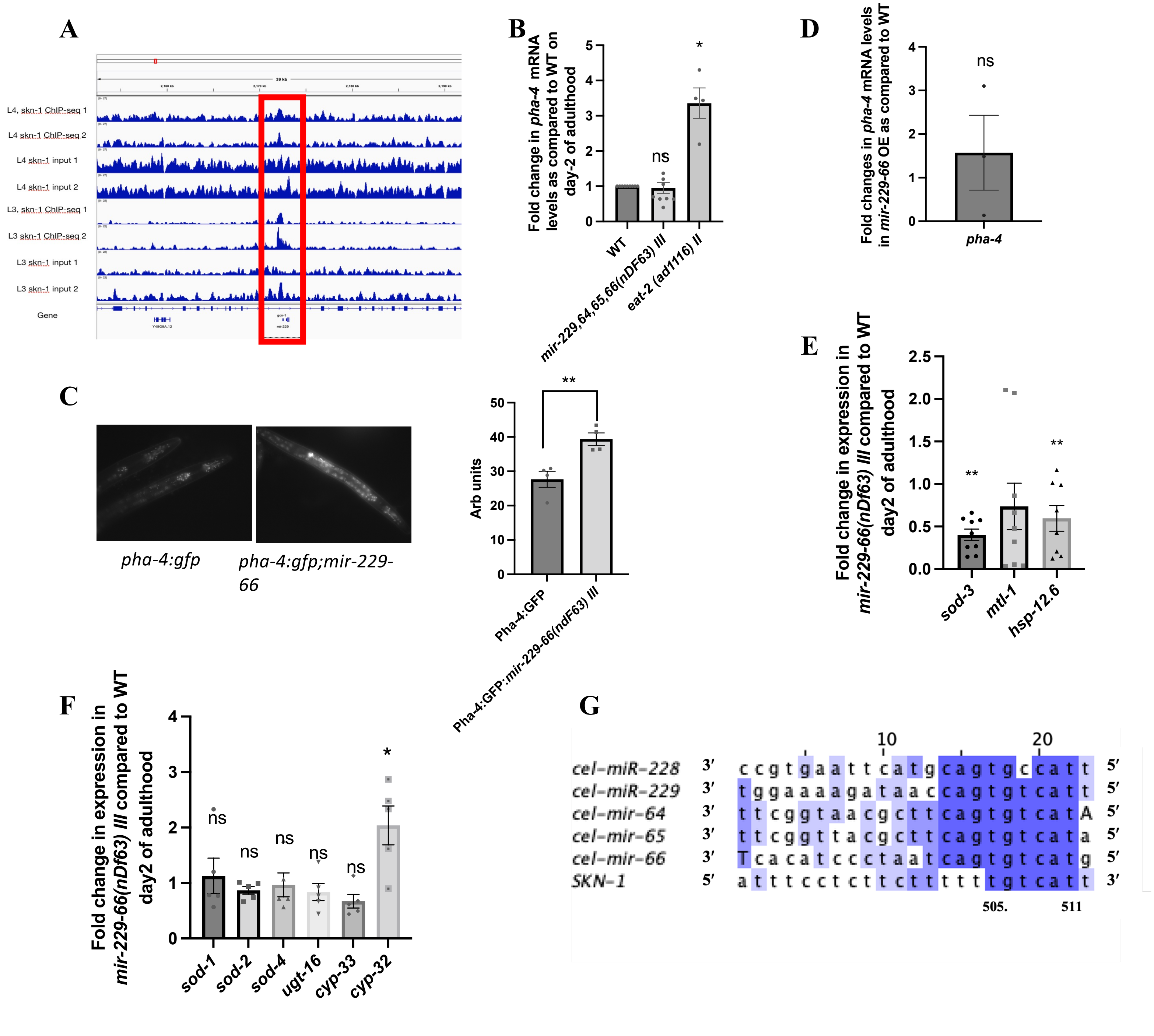
A
L4, skn-1 ChIP-seq 1
L4 skn-1 ChIP-seq 2
L4 skn-1 input 1
L4 skn-1 input 2
L3, skn-1 ChIP-seq 1
L3 skn-1 ChIP-seq 2
L3 skn-1 input 1
L3 skn-1 input 2
Gene
B
Fold change in pha-4 mRNA levels as compared to WT on day-2 of adulthood
WT
mir-229,64,65,66(nDF63) III
eat-2 (ad1116) II
ns
*
D
Fold changes in pha-4 mRNA levels in mir-229-66 OE as compared to WT
ns
pha-4
C
pha-4:gfp
pha-4:gfp;mir-229-66
Arb units
**
Pha-4:GFP
Pha-4:GFP:mir-229-66(ndF63) III
E
Fold change in expression in mir-229-66(nDf63) III compared to WT day2 of adulthood
sod-3
mtl-1
hsp-12.6
F
Fold change in expression in mir-229-66(nDf63) III compared to WT day2 of adulthood
sod-1
sod-2
sod-4
ugt-16
cyp-33
cyp-32
ns
*
G
cel-miR-228 3′ ccgtgaattcatgcagtgcatt 5′
cel-miR-229 3′ tggaaaagataacagtgtcatt 5′
cel-mir-64 3′ ttcggtaacgcttcagtgtcatA 5′
cel-mir-65 3′ ttcggttacgcttcagtgtcata 5′
cel-mir-66 3′ Tcacatccctaatcagtgtcatg 5′
SKN-1 5′ atttcctcttctttttgtcatt 3′
505.
511

Figure S5

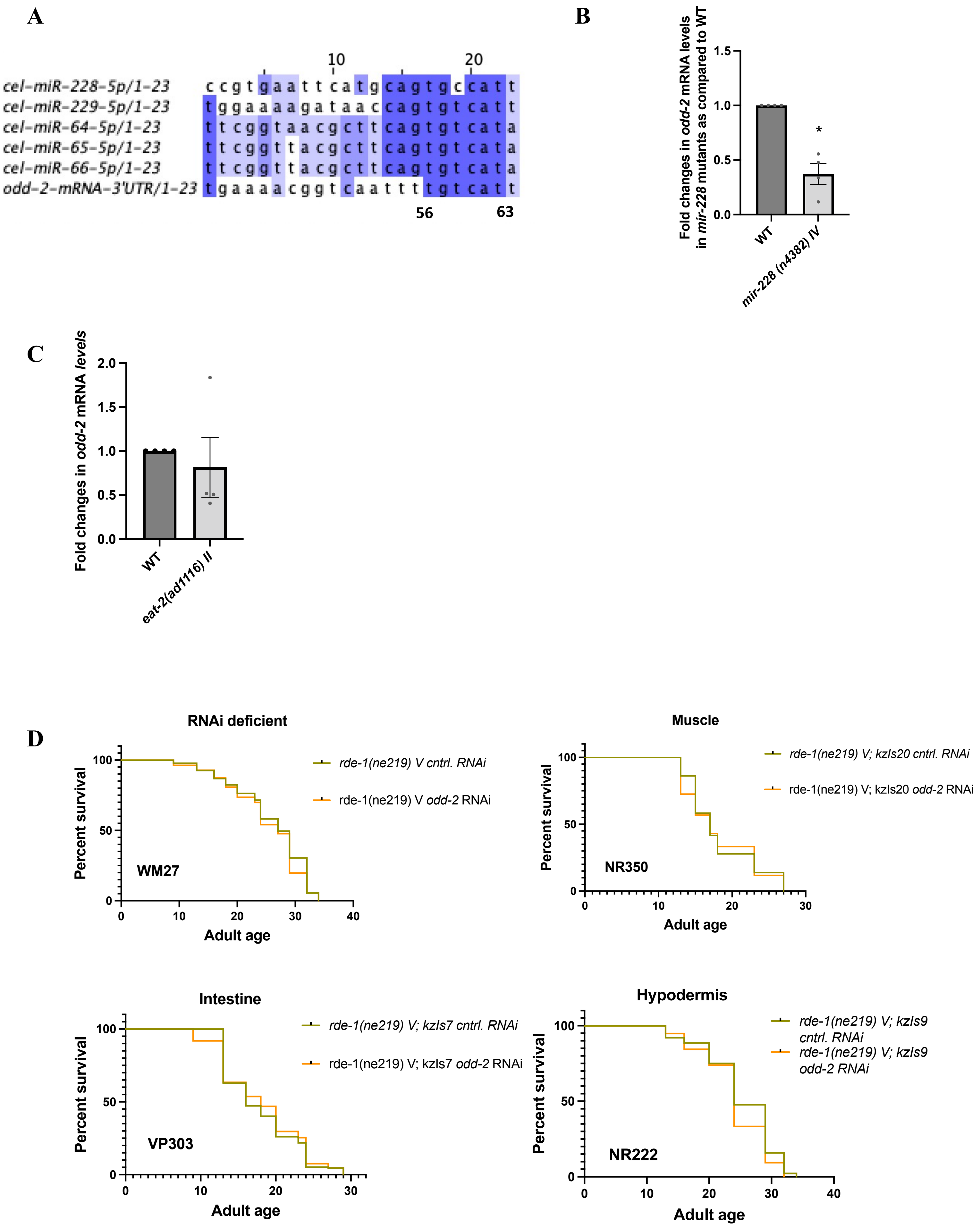
A
10
20
cel-miR-228-5p/1-23 ccgtgaattcatgcagtgccatt
cel-miR-229-5p/1-23 tggaaaagataacagtgtcatt
cel-miR-64-5p/1-23 ttcggtaacgcttcagtgtcata
cel-miR-65-5p/1-23 ttcggttacgcttcagtgtcata
cel-miR-66-5p/1-23 ttcggttacgcttcagtgtcata
odd-2-mRNA-3'UTR/1-23 tgaaaacggtcaatttttgtcatt
56
63
B
Fold changes in odd-2 mRNA levels in mir-228 mutants as compared to WT
1.5
1.0
0.5
0.0
*
WT
mir-228 (n4382) IV
C
Fold changes in odd-2 mRNA levels
2.0
1.5
1.0
0.5
0.0
WT
eat-2(ad1116) II
D
RNAi deficient
Percent survival
Adult age
WM27
rde-1(ne219) V cntrl. RNAi
rde-1(ne219) V odd-2 RNAi
Muscle
NR350
rde-1(ne219) V; kzIs20 cntrl. RNAi
rde-1(ne219) V; kzIs20 odd-2 RNAi
Intestine
VP303
rde-1(ne219) V; kzIs7 cntrl. RNAi
rde-1(ne219) V; kzIs7 odd-2 RNAi
Hypodermis
NR222
rde-1(ne219) V; kzIs9 cntrl. RNAi
rde-1(ne219) V; kzIs9 odd-2 RNAi

Figure S6

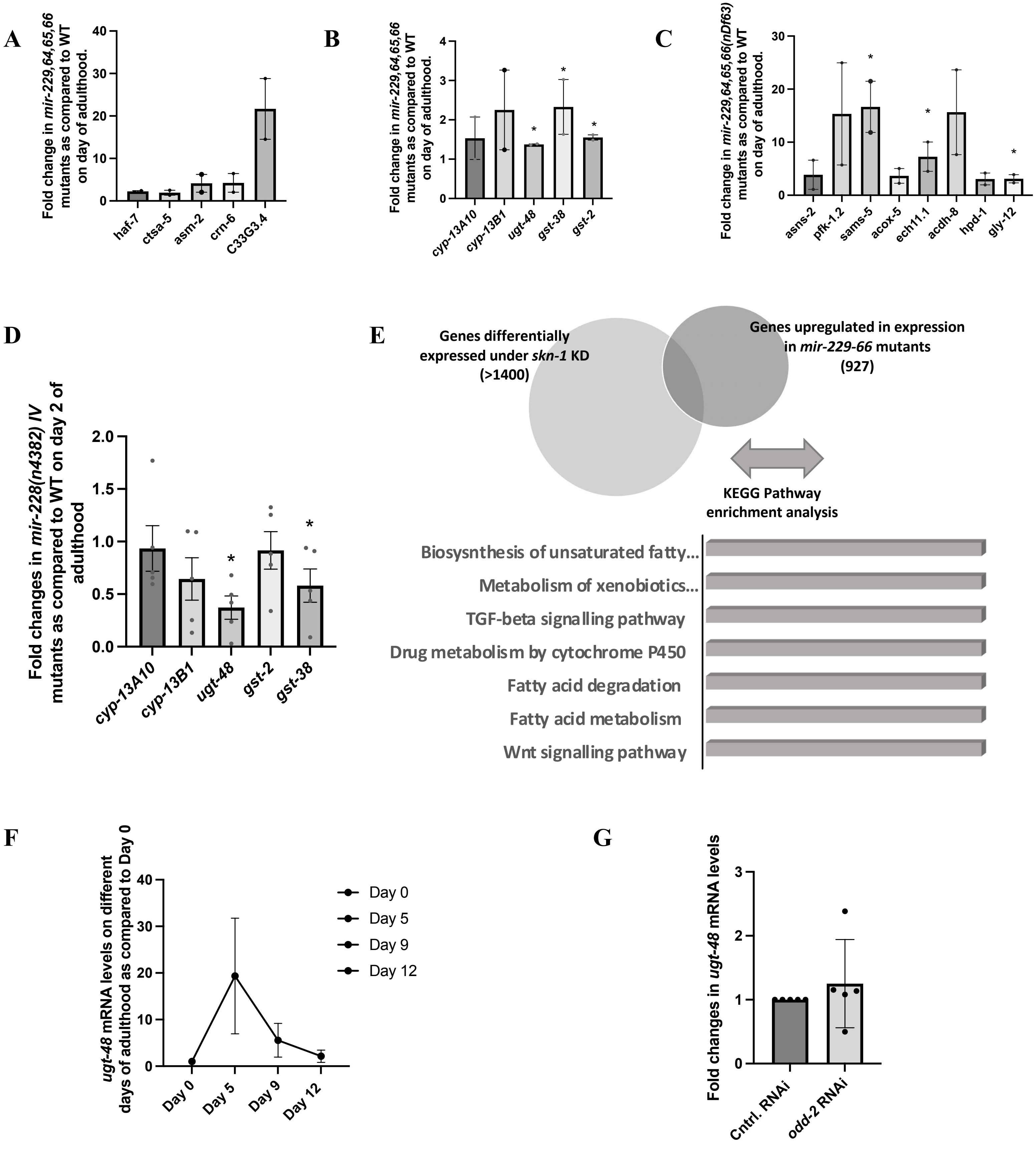
A
Fold change in mir-229,64,65,66 mutants as compared to WT on day of adulthood.
40
30
20
10
0
haf-7
ctsa-5
asm-2
crn-6
C33G3.4
B
Fold change in mir-229,64,65,66 mutants as compared to WT on day of adulthood.
4
3
2
1
0
*
cyp-13A10
cyp-13B1
ugt-48
gst-38
gst-2
C
Fold change in mir-229,64,65,66(nDf63) III mutants as compared to WT on day of adulthood.
30
20
10
0
asns-2
pfk-1.2
sams-5
acox-5
ech11.1
acdh-8
hpd-1
gly-12
D
Fold changes in mir-228(n4382) IV mutants as compared to WT on day 2 of adulthood
2.0
1.5
1.0
0.5
0.0
cyp-13A10
cyp-13B1
ugt-48
gst-2
gst-38
E
Genes differentially expressed under skn-1 KD (>1400)
Genes upregulated in expression in mir-229-66 mutants (927)
KEGG Pathway enrichment analysis
Biosysnthesis of unsaturated fatty...
Metabolism of xenobiotics...
TGF-beta signalling pathway
Drug metabolism by cytochrome P450
Fatty acid degradation
Fatty acid metabolism
Wnt signalling pathway
F
ugt-48 mRNA levels on different days of adulthood as compared to Day 0
40
30
20
10
0
Day 0
Day 5
Day 9
Day 12
G
Fold changes in ugt-48 mRNA levels
3
2
1
0
Cntrl. RNAi
odd-2 RNAi
